# Supplementary material for: Mental chronometry in big noisy data
Source: PLoS One. 2022 Jun 8;17(6):e0268916. doi: 10.1371/journal.pone.0268916 (PMC9176764; doi:10.1371/journal.pone.0268916)
Supplement: S1 Table — Additionally, data fitted with a Gaussian process regression model, upsampled to 1 MHz (Peak Fitted) are presented. Data are presented for all ERP components of interest and for 1/2, 1/3, 1/4, and 1/5 splits of the alternate-draw data set. (DOCX) [file pone.0268916.s001.docx]

S1 Table. Split-reliabilities (Spearman-Brown corrected) for peak latencies, fractional area latencies in individual averages and in jackknifed data. Additionally, data fitted with a Gaussian process regression model, upsampled to 1 MHz (Peak Fitted) are presented. Data are presented for all ERP components of interest and for 1/2, 1/3, 1/4, and 1/5 splits of the alternate-draw data set.

|  |  | Peak Latency | | Fractual Area | | JackKnife Peak Latency | | JackKnife  Peak (fitted) | | JackKnife Fractual Area | |
| --- | --- | --- | --- | --- | --- | --- | --- | --- | --- | --- | --- |
|  |  | min | max | min | max | min | max | min | max | min | max |
| P1 | 1/2 | **0.77** |  | **0.85** |  | NaN |  | **0.90** |  | **0.93** |  |
|  | 1/3 | 0.63 | **0.70** | **0.75** | **0.78** | NaN | NaN | **0.80** | **0.83** | **0.87** | **0.87** |
|  | 1/4 | 0.42 | 0.61 | 0.68 | **0.77** | 0.12 | 0.12 | **0.73** | **0.77** | **0.79** | **0.83** |
|  | 1/5 | 0.46 | 0.55 | 0.64 | **0.71** | NaN | NaN | 0.65 | **0.73** | **0.75** | **0.80** |
|  |  |  |  |  |  |  |  |  |  |  |  |
| N1 | 1/2 | **0.81** |  | **0.91** |  | NaN |  | **0.91** |  | **0.96** |  |
|  | 1/3 | 0.67 | 0.69 | **0.81** | **0.85** | 0.26 | 0.26 | **0.81** | **0.82** | **0.92** | **0.92** |
|  | 1/4 | 0.58 | 0.66 | **0.73** | **0.82** | 0.02 | 0.02 | **0.76** | **0.78** | **0.89** | **0.90** |
|  | 1/5 | 0.57 | 0.67 | 0.69 | **0.77** | 0.13 | 0.13 | 0.66 | **0.72** | **0.84** | **0.88** |
|  |  |  |  |  |  |  |  |  |  |  |  |
| P2 | 1/2 | **0.74** |  | **0.92** |  | NaN |  | **0.90** |  | **0.97** |  |
|  | 1/3 | **0.71** | **0.75** | **0.91** | **0.93** | NaN | NaN | **0.82** | **0.83** | **0.95** | **0.95** |
|  | 1/4 | 0.62 | 0.68 | **0.83** | **0.87** | 0.00 | 0.35 | **0.74** | **0.76** | **0.92** | **0.93** |
|  | 1/5 | 0.53 | 0.70 | **0.77** | **0.86** | NaN | NaN | 0.68 | **0.72** | **0.91** | **0.92** |
|  |  |  |  |  |  |  |  |  |  |  |  |
| N2 | 1/2 | **0.82** |  | **0.93** |  | 0.30 |  | 0.41 |  | **0.97** |  |
|  | 1/3 | **0.70** | **0.76** | **0.86** | **0.90** | 0.05 | 0.46 | 0.08 | 0.27 | **0.93** | **0.94** |
|  | 1/4 | 0.65 | **0.74** | **0.83** | **0.88** | 0.06 | 0.57 | 0.13 | 0.58 | **0.89** | **0.92** |
|  | 1/5 | 0.55 | **0.70** | **0.78** | **0.84** | 0.02 | 0.21 | 0.01 | 0.30 | **0.88** | **0.90** |
|  |  |  |  |  |  |  |  |  |  |  |  |
| P3 | 1/2 | **0.78** |  | **0.88** |  | 0.14 |  | **0.76** |  | **0.97** |  |
|  | 1/3 | 0.69 | **0.71** | **0.82** | **0.83** | 0.03 | 0.03 | 0.57 | 0.61 | **0.94** | **0.95** |
|  | 1/4 | 0.54 | 0.66 | **0.74** | **0.79** | 0.02 | 0.36 | 0.50 | 0.56 | **0.92** | **0.93** |
|  | 1/5 | 0.43 | 0.61 | 0.66 | **0.78** | 0.01 | 0.22 | 0.33 | 0.45 | **0.88** | **0.91** |
