# Supplementary material for: Mental chronometry in big noisy data
Source: PLoS One. 2022 Jun 8;17(6):e0268916. doi: 10.1371/journal.pone.0268916 (PMC9176764; doi:10.1371/journal.pone.0268916)
Supplement: S2 Table — (DOCX) [file pone.0268916.s002.docx]

S2 Table. Internal consistency (Cronbachs Alpha), data quality (Standardized Measurement Error = SME), and the effects of data pruning for the 1/5 data split for all measures retrieved and all ERP components of interest.

|  | Peak Latency | Fract | PeakJK | PeakFitJK | FractJK |
| --- | --- | --- | --- | --- | --- |
| **P1** | **0.81** | **0.89** | 0 | **0.90** | **0.93** |
| SME_P1 | 5.13 | 8.56 | 43.68 | 5.17 | 6.76 |
| **Pr_P1** | **0.81** | **0.91** | 0 | **0.91** | **0.95** |
| Pr_SME_P1 | 4.97 | 3.53 | 19.46 | 2.19 | 2.81 |
|  |  |  |  |  |  |
| **N1** | **0.86** | **0.92** | 0.03 | **0.90** | **0.96** |
| SME_N1 | 5.41 | 8.31 | 56.34 | 13.03 | 8.28 |
| **Pr_N1** | **0.88** | **0.94** | 0 | **0.91** | **0.97** |
| Pr_SME_N1 | 5.14 | 3.37 | 24.78 | 5.61 | 3.53 |
|  |  |  |  |  |  |
| **P2** | **0.85** | **0.94** | NaN | **0.90** | **0.98** |
| SME_P2 | 10.42 | 13.56 | 0.55 | 11.01 | 7.74 |
| **Pr_p2** | **0.89** | **0.97** | NaN | **0.90** | **0.98** |
| Pr_SME_P2 | 8.47 | 4.99 | 0.24 | 4.76 | 3.28 |
|  |  |  |  |  |  |
| **N2** | **0.87** | **0.94** | 0.07 | 0.06 | **0.97** |
| SME_N2 | 10.02 | 13.15 | 247.69 | 376.69 | 13.52 |
| **Pr_N2** | **0.90** | **0.96** | 0.17 | 0.45 | **0.97** |
| Pr_SME_N2 | 9.14 | 5.25 | 66.27 | 90.02 | 5.72 |
|  |  |  |  |  |  |
| **P3** | **0.83** | **0.92** | 0.27 | **0.71** | **0.97** |
| SME_P3 | 22.26 | 29.74 | 129.99 | 48.46 | 22.45 |
| **Pr_P3** | **0.86** | **0.94** | 0.30 | **0.74** | **0.97** |
| Pr_SME_P3 | 20.45 | 12.07 | 57.23 | 20.61 | 9.57 |
